# Port site recurrence after laparoscopic radical nephrectomy: a case report

**DOI:** 10.1186/s13256-017-1319-y

**Published:** 2017-06-08

**Authors:** Kota Shimokihara, Takashi Kawahara, Daiji Takamoto, Taku Mochizuki, Yusuke Hattori, Jun-ichi Teranishi, Yasuhide Miyoshi, Sawako Chiba, Hiroji Uemura

**Affiliations:** 1 Departments of Urology and Renal Transplantation, Yokohama City University Medical Center, Yokohama, Japan; 2 Department of Diagnostic Pathology, Yokohama City University Medical Center, Yokohama, Japan

**Keywords:** Port site metastasis, Port site recurrence, Laparoscopy

## Abstract

**Background:**

Due to the recent development of laparoscopic devices, laparoscopic radical nephrectomy is the standard procedure for localized renal cell carcinoma. However, some studies have reported postoperative port site metastasis in several cancers.

**Case presentation:**

A 68-year-old Asian-Japanese man was referred to our hospital for a further examination of his right renal tumor in 2009. Due to a clinical suspicion of renal cell carcinoma, laparoscopic nephrectomy was performed. The histopathological diagnosis was clear cell renal cell carcinoma. Follow-up computed tomography revealed a mass between the internal oblique muscle of his abdomen and the transverse muscle of his abdomen in 2014. The tumor size gradually increased, and positron emission tomography-computed tomography revealed the accumulation of fludeoxyglucose in that tumor with maximum standardized uptake value of 2.7. Based on these findings, port site recurrence was suspected, and tumor resection was performed in 2017. The pathological diagnosis was metastatic clear cell renal cell carcinoma.

**Conclusions:**

Here we report a rare case of port site metastasis that was successfully treated 7 years after laparoscopic nephrectomy.

## Background

Due to the recent development of laparoscopic devices, laparoscopic radical nephrectomy is the standard procedure for localized renal cell carcinoma. However, some studies have reported postoperative port site metastasis in gynecologic oncology [1], hepatocellular carcinoma [2–4], renal pelvis carcinoma [5], prostate cancer [6], and gallbladder carcinoma [7]. Here we report a rare case of port site metastasis that was successfully treated 7 years after laparoscopic nephrectomy.

## Case presentation

A 68-year-old Asian-Japanese man was referred to our hospital for a further examination of his right renal tumor in 2009. He had no remarkable family or medical history, except for hyperuricemia and overactive bladder. Due to a clinical suspicion of renal cell carcinoma, laparoscopic nephrectomy was performed. The histopathological diagnosis was clear cell renal cell carcinoma, sized 30 × 25 × 24 mm.

**Fig. 1:**
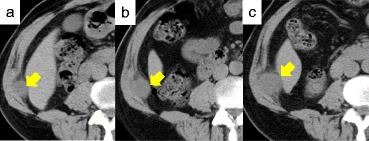
Computed tomography image of the right mass in **a**) October 2014, **b**) October 2015, and **c**) October 2016 (arrow; tumor)

**Fig. 2:**
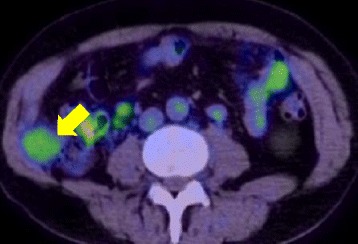
Positron emission tomography-computed tomography image of the mass (*arrow*)

**Fig. 3:**
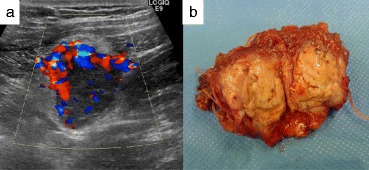
**a** Ultrasonography image. **b** Macroscopic findings for the tumor

Follow-up computed tomography (CT) revealed a mass between the internal oblique muscle of his abdomen and transverse muscle of his abdomen in 2014. The tumor size gradually increased (Fig. 1). Positron emission tomography (PET)-CT revealed an accumulation of fludeoxyglucose (FDG) in the tumor with a maximum standardized uptake value (SUVmax) of 2.7 (Fig. 2). Ultrasonography showed a hypervascular lesion in that tumor (Fig. 3). Based on these findings, port site recurrence was suspected, and tumor resection was performed in 2017.

The tumor was located just below the laparoscopic port site on ultrasonography and was 3 cm in diameter. A 5-cm skin incision was performed, and the fascia of his obliquus externus abdominis muscle was cut. The tumor was resected with his internal oblique and transverse abdomen muscles. We set the tumor margin at 5 mm using ultrasonography. Since the tumor adhered to his peritoneum, it was resected with the peritoneum.

**Fig. 4:**
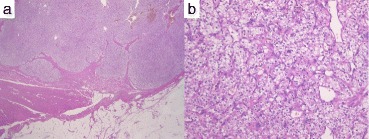
Microscopic findings (hematoxylin and eosin). **a** ×12.5; **b** ×200

The tumor was composed of cells with clear cytoplasm and contained solid cell nests that were separated by a prominent sinusoidal vascular network (Fig. 4). The pathological diagnosis was metastatic clear cell renal cell carcinoma.

## Discussion

Laparoscopic procedures have developed rapidly over the past decade. In T1 and T2 renal cell carcinoma, laparoscopic surgery now shows the same survival and recurrence rates as open surgery. Port site metastasis is sometimes reported, especially in cases of gallbladder cancer (7 to 17%), colorectal cancer (5%), and gynecological cancer (4%) [8, 9].

In urothelial cancers, port site recurrence has been reported in a total of 13 cases, as reviewed by Micali *et al*. in 2004: the incidence was 0.12% (13 of 10,912) [10, 11]. Of these 13 cases, there were four metastatic adrenal carcinomas, four urothelial carcinomas, three nephron-ureterectomy cases of upper urothelial carcinoma, one case of retroperitoneal lymph node resection for testicular cancer, and one case of lymph node resection for penile cancer. Their study included 2604 laparoscopic radical nephrectomy cases and 555 laparoscopic partial nephrectomy cases, but no port site recurrence was observed [11]. Recently, Song *et al*. reported port site metastasis in a total of 16 cases [12]. Our case was initially diagnosed as pT1a, G1 > G2 clear cell renal cell carcinoma and carried quite a low risk compared to the previous report.

According to the pertinent literature, most reported cases were margin-positive cancer, no-wrap removal, or ruptured tumors. In addition, among these cases, most were high-grade carcinoma (Fuhrman grade 3). Compared with previously reported cases, our case was low-grade carcinoma and had no technical problems, and the tumor was removed using a collecting bag without rupture. Three candidate mechanisms have been proposed, as follows: (1) The cancer cells attached to the surgical devices adhere to the laparoscopic port, gradually proliferating at the port site, (2) carbon dioxide gas induces immunosuppressive condition at the site, and (3) cancer tumors are introduced to the port site via carbon dioxide gas and attach and adhere to the site [13]. Yasuda *et al*. hypothesized that most cancer cells may have originated from sealing devices [14]. To prevent the attachment of cancer cells, collecting bags are recommended. In cases with ascites, laparoscopic surgery is not recommended [8].

For port site metastasis renal cell carcinoma, surgical resection, medical treatment, and radiotherapy have been reported. However, no consensus on the optimum treatment has been achieved. In general, for metastatic renal cell carcinoma, when possible, complete surgical resection is suggested as the gold standard treatment. For non-organ metastatic port site metastasis, a long recurrence-free survival has been reported [15].

It is important to consider the possibility of Schloffer tumors in the differential diagnosis for port site metastasis. Schloffer tumors develop due to a reaction against a foreign body, such as surgical stitches, at the site of incision. This benign inflammatory tumor builds up from a few months to a few years after surgery. PET-CT is occasionally performed to differentiate port site metastasis from Schloffer tumors [15]. In the present case, due to the rare incidence of port site metastasis after radical nephrectomy, CT, ultrasonography, and PET-CT were performed to detect the nature of the tumor. The tumor showed marked size progression on CT, a hypervascular nature on ultrasonography, and the uptake of FDG on PET-CT; tumor resection was performed without a fine needle biopsy due to the hypervascular nature of the tumor.

## Conclusions

Here we reported a rare case of port site metastasis that was successfully treated 7 years after laparoscopic nephrectomy.
